# Susceptibility weighted imaging can be a sensitive sequence to detect brain damage in neonates with kernicterus: a case report

**DOI:** 10.1186/s12883-023-03125-6

**Published:** 2023-03-11

**Authors:** Maarten Lequin, Floris Groenendaal, Jeroen Dudink, Paul Govaert

**Affiliations:** 1 UMC Utrecht: Universitair Medisch Centrum, Utrecht, Netherlands; 2 ZNA Middelheim, Antwerp, Belgium

**Keywords:** Susceptibility weighted imaging, SWI, Kernicterus, Neonatal, MRI

## Abstract

**Background:**

Kernicterus in the acute phase is difficult to diagnose. It depends on a high signal on T1 at the globus pallidum and subthalamic nucleus level. Unfortunately, these areas also show a relatively high signal on T1 in neonates as an expression of early myelination. Therefore, a less myelin-dependent sequence, like SWI, may be more sensitive to detecting damage in the globus pallidum area.

**Case presentation:**

A term baby developed jaundice on day three following an uncomplicated pregnancy and delivery. Total bilirubin peaked at 542 μmol/L on day four. Phototherapy was started, and an exchange transfusion was performed. ABR showed absent responses on day 10. MRI on day eight demonstrated abnormal high signal globus pallidus on T1w, isointense on T2w, without diffusion restriction, and high signal on SWI at globus pallidal and subthalamus level and phase image at globus pallidal level. These findings were consistent with the challenging diagnosis of kernicterus. On follow-up, the infant presented with sensorineural hearing loss and had a work-up for cochlear implant surgery. At 3 months of age, the follow-up MR shows normalization of the T1 and SWI signals and a high signal on T2.

**Conclusions:**

SWI seems more sensitive to injury than the T1w and lacks the disadvantage of the T1w sequence, where early myelin confers a high signal.

## Background

Kernicterus, a clinicopathological term that describes the acute neonatal encephalopathy associated with brain toxicity due to unbound unconjugated bilirubin, stands out among other neonatal brain disorders by a characteristic multiregional damage pattern [1–4]: globus pallidus, nucleus subthalamicus, geniculate nuclei, dentate nuclei, inferior olive, nucleus gracilis and cuneatus, hippocampus, corpora mammillaria, nucleus ruber, substantia nigra, cranial nerve nuclei, colliculi, vermis cerebelli. A pallido-subthalamic pattern of injury is distinct from the thalamo-striate pattern that is typical of acute total asphyxia [2, 5]. The main effects of bilirubin on neurons are decreased oxygen consumption and increased release of calcium and caspase 3, resulting in apoptosis [6]. A similar pattern is observed in oligodendrocytes, with increased apoptosis, oxidative stress, and reduced myelin synthesis. Microglia react to injury associated with bilirubin by releasing proinflammatory cytokines and metalloproteinase activity as cells manifest the phagocytic phenotype. Once the intracellular concentration of bilirubin exceeds a toxic threshold, a metabolic cascade leads to neurotoxicity. This means that the signature of kernicterus in the brain in the acute stage is region specific and characteristic of apoptosis. The neuronal swelling that alters diffusion characteristics in MR sequences in the acute phase after asphyxia is typically absent in kernicterus. However, more subtle alterations have been reported in the dentate to thalamus connections [7]. Globus pallidus and nucleus subthalamicus are hyperintense on T1 weighted MRI in the acute stage, but this is only a gradient of the increased signal compared to normal and, therefore, not distinctive enough to be sure about the extent of neuronal death; after several weeks, only a fine fibrillary astrogliotic scar permanently marks globus pallidus on T2 weighted MRI [8]. Many but not all infants with an acute encephalopathic presentation (day 1–2: stupor, hypotonia, poor feeding, apnea, convulsions; day 3–7: extensor hypertonia, fever, high pitched cry, tongue protrusion, 2w-3 m: hypotonia) go on to develop the typical outcome with dyskinetic cerebral palsy (extrapyramidal syndrome, vertical gaze palsy), dental dysplasia and hearing loss based on injury to the brainstem nuclei of the auditory system. Often, therefore, confirmation of the existence and extent of nuclear damage (“kern” icterus) is sought in acute neonatal MRI [9]. Due to uncertain descriptions of the extent and severity of the neuronal injury, kernicterus is still a challenging disorder in neonatal imaging.

## Case presentation

Our patient, a Caucasian male infant, was born via primary cesarean section at 41 2/7 weeks of gestation. Antenatal imaging and serology were normal, and there was no family history of neonatal jaundice. The infant was born in good condition with Apgar scores of 9 and 10 at 1 and 5 in, respectively, with a birth weight of 3705 g (p45). The patient was discharged from the local hospital in good clinical condition with no signs of jaundice on day two of life. After an uneventful day at home with the parents, where the baby was bottle-fed, the parents noticed their baby was jaundiced on day three after birth, and the midwife referred the patient to the hospital on day 4. Laboratory findings on admission to the local hospital revealed significant hyperbilirubinemia with a total bilirubin of 573 μmol/L and direct bilirubin of 38 μmol/L. (further lab: pH 7,44, PCO2 38 mmHg, pO2 95 mmHg, Act bic 25,6 mmol/L, BE 1 mmol/L, Sodium 139 mmol/L, Potassium 3,4 mmol/L, Ca-ion 1,22 mmol/L, Gluc 5,2 mmol/L, AF 152 U/L, yGT 34 U/L, ASAT 56 U/L, ALAT 14 U/L, LDH 922 U/L, Hb 6,9 mmol/L, Leucocytes 17,9 × 10^9/L, Trombocytes 346 × 10^9/L, PT 11,3 sec, Blood type A, Rhesus D pos, Direct Coombs positive.) The blood type of the mother was A and Rhesus positive. In his admission to the local hospital, he was presented with a weak general condition with a progressive opisthotonus. Triple phototherapy was started, and the patient was transferred to our NICU for an exchange transfusion. The patient had a successful exchange transfusion after arrival at our tertiary care center (posttransfusion total bilirubin of 223 μmol/L and direct bilirubin of 17 μmol/L). However, after the exchange transfusion, the patient had both clinical (apnea) and electrophysiological seizures, for which he was intubated and ventilated. The seizures were treated with phenobarbitone (2 × 10 mg/kg iv). After treatment, the (a) EEG returned to a normal background pattern, and the patient could be detubated. An ultrasound scan showed no significant abnormalities (e.g., hemorrhages).

 The ALGO and BERA showed no responses. Whole exome sequencing and variant analysis did not show protein-coding exons for hereditary hemolytic anemia.

**Fig. 1:**
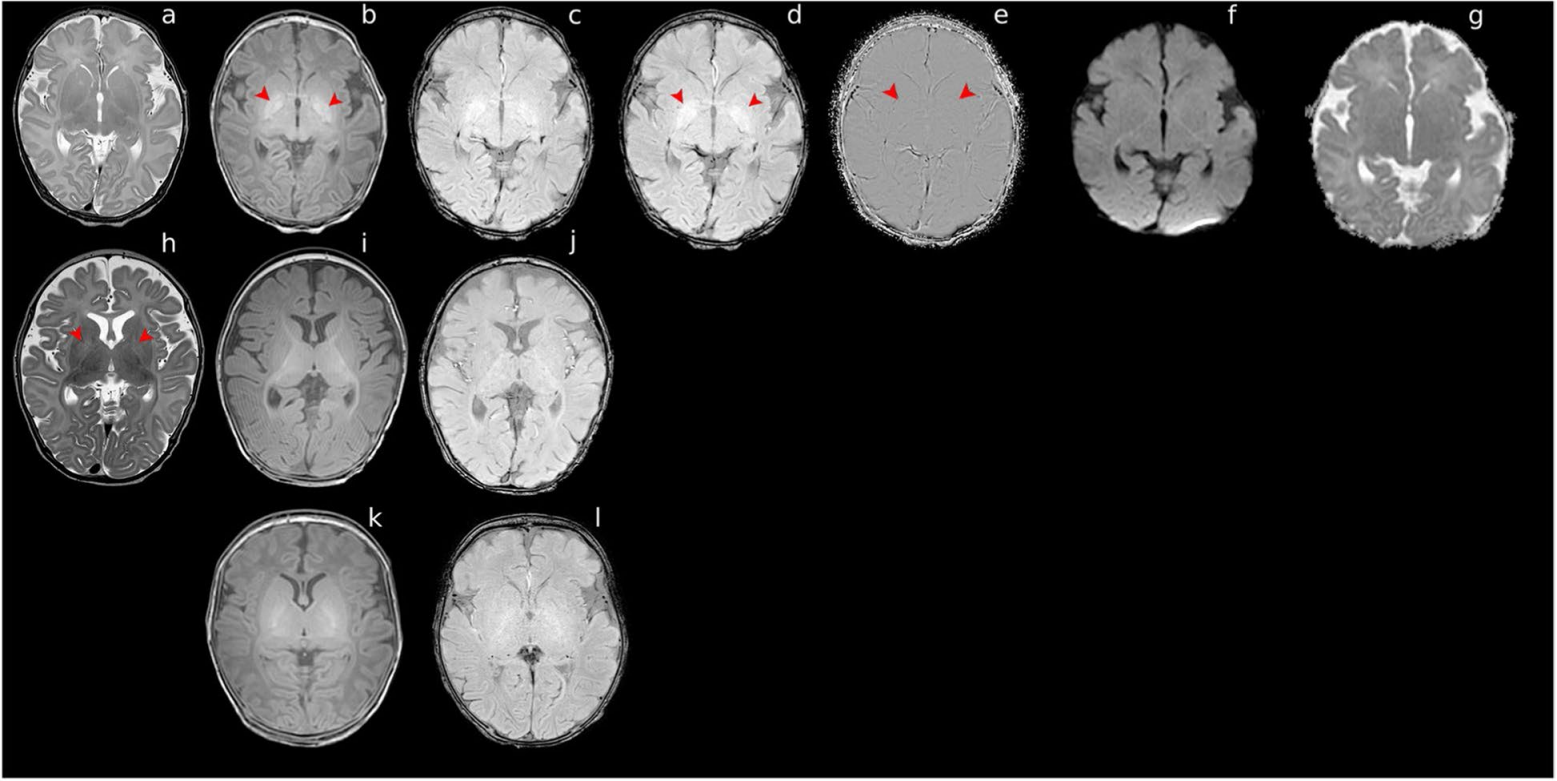
MR images in an 8-day-old term infant. Upper row: Axial T2 (TR 6282 ms, TE 120 ms, slice thickness 2 mm, no gap) shows no high signal intensity at globus pallidus level (**a**). Axial 3D T1(TR 9,38 ms, TE 4,6 ms, slice thickness 2 mm) shows a high signal in the basal ganglia (**b**). SWI (spoiled T1 enhanced with TR 31 ms, TE first 7,2 ms, slice thickness 2 mm) shows a high signal at the subthalamus (**c**) and globus pallidus level (**d**). The Phase image at the level of globus pallidus level shows no abnormal signal compatible with calcifications or blood products (**e**) Axial DWI, b 1000 and ADC map (TR 4998 ms, TE 102 ms, slice thickness 3 mm) shows no abnormal increased signal in basal ganglia (**f**, **g**). Middle row: At three months of age, the followup MR shows normalization of the signal on T1 and SWI (**i** and **j**) and a high T2 signal at the globus pallidal level (**h**). Lower row: Term neonate as control scanned in the first week of life. A normal signal at the basal ganglia on Axial 3D T1(TR 9,38 ms, TE 4,6 ms, slice thickness 2 mm) (**k**) and SWI (spoiled T1 enhanced with TR 31 ms, TE first 7,2 ms, slice thickness 2 mm) (**l**)

MR images in an 8-day-old term infant show abnormal high signal intensity at the globus pallidus level on SWI (Fig. 1).

## Discussion and conclusions

Kernicterus is a rare and serious condition that gives rise to acute encephalopathy in the neonatal period and can cause long-lasting negative neurocognitive outcomes, including hearing loss and dyskinetic cerebral palsy [10]. Close monitoring of bilirubin levels and timely treatment when bilirubin levels reach nationally defined treatment levels can prevent kernicterus. However, the clinical presentation of severe hyperbilirubinemia may not always be typical [3]. It can be missed, and sometimes kernicterus can occur with bilirubin levels below those stated in the national treatment guidelines [11]. The MRI findings in the disease’s acute phase may aid in diagnosis. To date, neuroradiologists have had to rely on high-intensity signals (T1-weighted) at the level of the globus pallidum and subthalamic nuclei [8, 9]. However, determining kernicterus may not be straightforward due to the intrinsic T1-weighted hyperintensity signal in these areas, which is attributed to early myelination.

Also, the extent of the neuronal injury could be hampered due to the inherent high signal on T1. Since our MR protocol for scanning neonates with encephalopathy includes an SWI sequence, we noticed an abnormally high signal at the globus pallidum region and less at the subthalamic region in the case of kernicterus. The signal is more conspicuous than the high signal on the T1 sequence.

Moreover, these involved areas are not as high on SWI in normal neonatal controls, making it easier to recognize the damaged regions and may better delineate their extent and severity. Knowing that the main effects of bilirubin on neurons are decreased oxygen consumption and increased release of calcium and caspase 3, resulting in apoptosis [6], this may result in a high signal on the SWI sequence. This is important because other sequences lack discriminating power, like the commonly used sequences, DWI, and T2 sequences in the acute phase. The high signal on the magnitude image of the SWI sequence of our case is not due to a cluster of calcifications, looking at the normal signal on the phase image of the SWI sequence. Another explanation of the high signal in the globus pallidal region could be the influx of cells or edema as a response to the injury, best detected by the SWI due to its small flip angle, around 17 degrees, making the SWI images spin-density-weighted [12]. When we had only the spin echo T2, the proton density image also showed a high signal at the globus pallidal region in kernicterus cases, suggesting increased proton density in these regions.

Also, MRS seems to give contradictory results, though Groenendaal et al. reported a lactate peak at the globus pallidum area in some kernicterus cases [13]. Our follow-up MR at 3 months of age shows normalization of the high signal on SWI and on T1, which has already been mentioned by Govaert et al. [8]. In conclusion, we speculate that a high signal on SWI at the globus pallidum region is a promising imaging biomarker for recognizing kernicterus damage in the acute phase.

## Data Availability

The data supporting this case’s findings are available at reasonable request from the corresponding author. The data are not publicly available due to privacy or ethical restrictions.
